# “Il Corpo Ritrovato”: Dermocosmetological Skin Care Project for the Oncologic Patient

**DOI:** 10.5402/2011/650482

**Published:** 2011-04-27

**Authors:** G. Fabbrocini, M. C. Romano, N. Cameli, M. Mariano, F. Pastore, M. C. Annunziata, C. Mazzella, Valerio De Vita, Maria Chiara Mauriello, G. Monfrecola

**Affiliations:** ^1^Division of Clinical Dermatology, Department of Systematic Pathology, University of Naples Federico II, Street Sergio Pansini 5, Naples 80133, Italy; ^2^ASL ROMA C and Division of Clinical Dermatology, University of Rome “Tor Vergata”, 00133 Rome, Italy; ^3^Surgery of Aesthetic Dermatology of San Gallicano Dermatological Institute (IRCCS), 00118 Rome, Italy

## Abstract

Neoplastic disease and its therapeutic options have a huge impact on the patient's quality of life from both the emotional and the working point of view. The project “Il Corpo Ritrovato” aims at creating an interdisciplinary network of physicians to improve the quality of life of the oncologic patient, focusing on such important aspects as dermocosmetological skin care but also on the evaluation of new therapeutic and diagnostic algorithms in order to make further progress in the field of prevention.

## 1. Introduction

Neoplastic disease and its therapeutic options have a huge impact on the patient's quality of life from both the emotional and the working point of view [1]. Sociological research has revealed that gender also plays an important role in the emotional reaction to the disease [2, 3]. When faced with a tumoral disease, men and women show significantly different reactions: as wives and mothers are more subject and vulnerable to stress, they present pluripathological situations more often than their partners and require more frequent medical care. 

Moreover, from a medical point of view, the short-term modifications of the skin are the most evident and crippling consequences of chemotherapies, above all from a psychological point of view. Although chemotherapy contrasts cancer, it often does not preserve good cells and organs, resulting in a series of strongly crippling cutaneous alterations.

In chemotherapy treatment of some tumors (uterus, ovary, breast), pharmacological damage is often potentiated by hormonal “corrections” that aggravate the situation by causing premature ageing compounded with the chronological and photo-induced process. This provokes serious discomfort in female patients because of the aesthetic modifications of their image.

The possibility to reduce hair loss and various nail alterations or to improve skin colour, texture, and quality with personalized cosmetic treatments, studied and structured for specific skin types, can provide a comforting support for female patients facing oncologic therapies.

We can also limit the negative effects of radiotherapy treatment by reducing cellular damage with the aid of applications, before and after the radiation session, of specific therapeutic and preventive aids. It is also important to contain any destructive damage and to address the prevention and care of cicatricial results.

These considerations have led to the birth of the project “Il Corpo Ritrovato” and a scientific board (president: pucci maria concetta romano; vice-president: gabriella fabbrocini; secretary: Norma Cameli) with Lega Tumori (LILT) and under the aegis of the Italian Ministry for Equal Opportunities. The project aims at creating an interdisciplinary network of physicians to improve the quality of life of the oncologic patient, focusing on such important aspects as dermocosmetological skin care but also on the evaluation of new therapeutic and diagnostic algorithms in order to make further progress in the field of prevention.

## 2. Chemotherapy and Skin

Chemotherapy causes several adverse events acting against mucosa and cutis (Table 1) and adnexa, and many such reactions are not known because of the continuous innovation in this field [4]. The antineoplastic drug targets are keratinocyte mitotic activity in the epidermis and fibroblasts, collagen fibres, and amorphous material in the derma.

At adnexa level, sudoriparous and sebaceous gland regulation is affected and hair trophism is involved. In the pigmentary cells, there is melanocyte synthesis inhibition and/or melanin anarchic synthesis. Keratinocytes lose their form, mobility, cohesion, and division capability, resulting in slower epidermis turnover. The reticular dermis reduces its thickness and diminishes its vascularization. The papillary dermis is characterized by matrix alterations with a reduction in collagen, elastic fibres, glycosaminoglycans, and water. Furthermore, the recovery capability after pressure is diminished (loose skin).

Moreover, iatrogenic and chronological ageing effects determine and increase wrinkles. Among the adnexa, the number and the functionality of sudoriparous and sebaceous glands are reduced with a consequential cutaneous dryness, pore dilatations, and appearance of skin papulose lesions. Hair follicles become terminal follicles.

In recent years, new classes of drugs used in oncology have determined numerous and increasingly frequent skin reactions. Such drugs include inhibitors of epidermal growth factor receptor (EGFr), which is largely expressed by basal keratinocytes, sebaceous cells, and endothelial cells [5]. 

**Figure 1:**
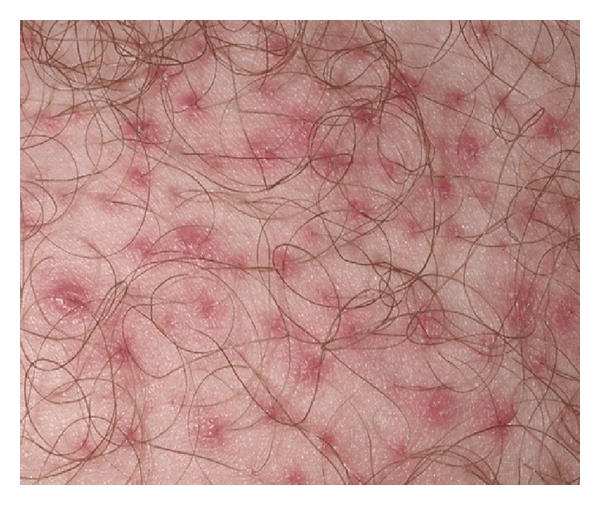
Papular-pustular follicular rash during gefitinib therapy.

The most frequently reported toxic cutaneous effect deriving from these drugs is the papulose pustulous follicle rash, which is defined as a form of acne since it involves above all the face's seborrhoeic areas, scalp, and chest, and less frequently the extremities and the back. Such an eruption appears during the first two weeks of treatment, accompanied by an extremely irritating pruritus and can be complicated by bacterial overinfections, albeit short lived. Its peculiar characteristic is the association of a typical sebaceous gland disease with a marked xerosis, indicating that the pathology protagonist is not the cutaneous adnexa but the keratinocyte itself (Figure 1).

Mucosa and cutis xerosis, varying from the light to the most marked forms with eczema and fissures, has so far shown a variable incidence from 12% to 35% in clinical trials [6], and it often represents one of the cutaneous parameters persistently influencing the patient's life quality.

**Figure 2:**
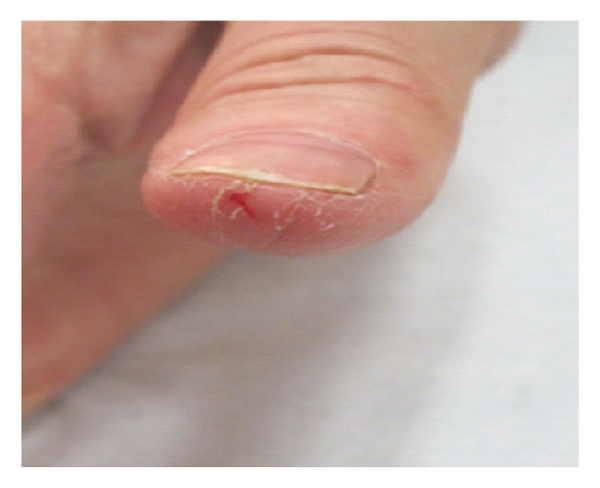
Fissures of finger tips during cetuximab therapy.

**Figure 3:**
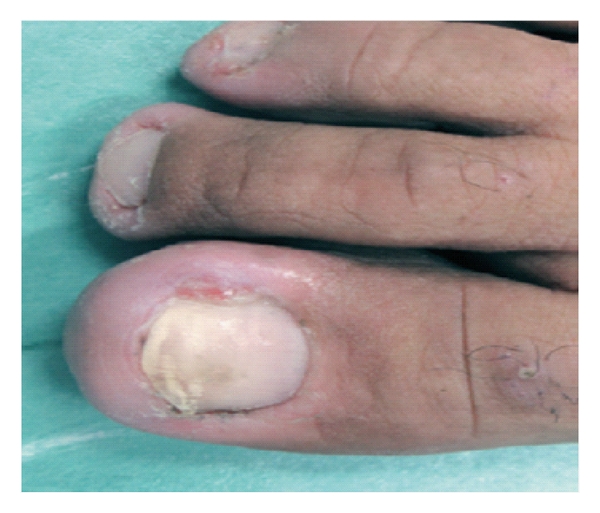
Pyogenic granuloma and nail infection during cetuximab therapy.

Onychopathologies (Figures 2 and 3), which are likewise long lasting and thus highly crippling, can be found in 10–20% of patients under oncotherapy and may appear above all as pigmentation alteration, onychodystrophies, paronychia, onychocryptosis, and consequential recidivated or recurrent onychomycosis.

The most widely recognized effect that stigmatizes and frightens the oncologic patient is alopecia induced by chemotherapic drugs. The incidence and extent of alopecia vary according to the antineoplastic drug most frequently employed, above all for taxanes, anthracyclines, and alkylating agents [7] (Table 2). It is generally a grave form of scalp alopecia characterized by dystrophic effluvium anagen that appears in 1 to 8 weeks from the commencement of chemotherapy, and it is usually reversible. However, cases of permanent alopecia caused by chemotherapy are known, associated above all with busulfan administration (50% of patients) and radiation in doses > 700 Gy [8].

Other terminal hairs (beard, lash, eyebrow, pubic, and axillary hairs) are interested by alopecia to varying degrees, according to the percentage of those in anagen phase at the beginning of the therapy and to the duration and posology of chemotherapic treatment. Moreover, other hair alterations may include isolated and slow hair growth, hair becoming frizzier and curlier, trichomegaly, that is, excessive growth of lash diameter and length, suggesting that the regulating mechanisms at the basis of these processes may have different pathogenetic origins.

Hypersensitivity reactions (urticaria, vasculitis, polymorphic erythema, Stevens-Johnson syndrome, etc.), flashings, and acral erythema, cutaneous pigmentation alteration, mucosa inflammation (oral and anal), and photosensitivity reactions are less frequent but no less important and require a careful study in order to establish the most adequate dermocosmetologic algorithm for each case.

## 3. Cutis and Radiotherapy

Nowadays, radiotherapy is a common therapeutic aid in oncology, one of the more common cutaneous toxic effects of which is radiodermatitis. Indeed, about 85% of the patients under radiotherapy develop skin manifestations which can be related to this and range from a moderate erythematosus rash to ulceration proper [9].

When this procedure is associated with a chemotherapic algorithm,* radiation recall *and *radiation enhancement* can add to the said skin lesions. Radiation recall is characterized by the presence of an inflammatory reaction on a previously irradiated area, after chemotherapic drug assumption. It takes place within a few hours or days when the chemotherapic drug is administered from 8 to 15 days after radiotherapy. It is an erythematous rash that can be associated in different ways to vesiculation, scaling, and itching.

*Radiation enhancement* is the enhancement of skin toxicity deriving from radiotherapy caused by chemotherapic drug administration, which may be contemporary or 7 days after radiotherapy. The drugs more frequently involved in such reactions are above all: bleomycin, doxorubicin, fluorouracil, hydroxyurea, 6-mercaptopurine, and methotrexate. From a clinical point of view, it appears as radiodermitis, with erythema, edema, vesicles, blisters, or erosions and in severe cases with tissue necrosis which, unlike radiation recall, can locally expand beyond the irradiated area.

Both manifestations present self-limitation over several months, but they are strongly crippling since they can survive in long-term sequels like cutaneous atrophy, telangiectasia, and fibrotic results.

## 4. Dermocosmetologic Algorithm

The identification and validation of guidelines to be used in the elaboration of the ad hoc dermocosmetologic algorithm for an oncologic patient represents an important supporting instrument in the correct care of such a patient.

Moreover, in the pharmacological management of skin toxicity reactions, there is a series of recommendations that are quite empiric and not validated by evidence. They are not yet used in daily clinic practice, do not involve specific cosmetologic aspects, and do not offer any therapeutic advice specific to the patient. 

In any case, from the beginning of each treatment cycle, the patient under oncologic treatment should adopt a series of preventive behaviours and hygienic measures to counter collateral effects against cutis.

Our algorithm aims at keeping the various collateral effects under control, in terms of both prevention and therapeutic prescription.

### 4.1. Xerosis

 In order to avoid this annoying effect, it is necessary to pay attention both to cleansing and hydration. Detergents with few or no aggressive surfactants and foaming substances should be preferred (sodiolauril-sulfate, sodiolauriletere-sulfate) and an “affinity” cleansing should be preferred.

Therefore, W/O emulsions with lipid component characterized by both natural vegetable fats (karité, wheat germ oil, jojoba, avocado) and synthetic ones (caprylic capric triglyceride) should be recommended; on the other hand, cosmetics with a large percentage of by-products of hydrocarbons (petrolatum, paraffin wax, vaseline) and silicones (cyclomethicone, dimethicone, cyclopentasiloxane, cyclohexasiloxane) which are, at present, considered carcinogens of series II, even if not subjected to any regulation on the allowed percentage, should be avoided.

The same can be said for the choice of cosmetics aiming to re-establish hydration and to oppose the oxidative effect of drug therapy.

The appropriate hydrating substance should contain selected and active agents: unsaponifiable substances (karité, jojoba, olive, palm), aloe, niacinamide (vit. B3), tocopherols, tocotrienols, ceramides, and gamma oryzanol.

The absence or minor presence of petrolatum and silicones is important because hydration is not re-established by mechanisms of maceration of the stratum corneum.

Exfoliating and irritating creams, for example glycolic acid, alpha-hydroxy acids, and benzoyl peroxide or alcohol gel formulations, are not recommended in this particular period of skin fragility because of their drying and irritant power.

If the itch is not tolerated, we can administer anti-H1 antihistamines (cetirizine, loratadine, fexofenadine).

Major xerosis can cause ulcerations and fissures, for which we can recommend the use of 2% eosin in local applications together with creams or pastes based on vitamin E and zinc oxide. The choice of an ointment formulation (made from urea and not petrolatum), including that related to topical antibiotics, should be always privileged.

### 4.2. Nail Damage

The prevention of onychopathology can be aided by a special cooling glove [10], worn during the infusion, which reduces the local toxicity of drugs due to vasoconstriction induced by low temperature (up to- 25°/ -30°C).

 If there is nail infection, we should apply topical antiseptics to prevent overinfections as well as topic rifampicin and, if necessary, topical silver nitrate to reduce granulation tissue.

 In more serious cases, we can use oral antibiotics (doxycycline or minocycline) without any chemotherapy interruption and, when we suspect a bacterial or fungal overinfection, a cultural examination is important to establish the therapy.

### 4.3. Alopecia

Alopecia is certainly the most feared and crippling mucocutaneous effect caused by chemotherapy. The use of topical 2% minoxidil seems to give good results especially in the case of alopecia caused by taxanes and anthracyclines, but it does not prevent alopecia caused by doxorubicin [11]. Currently, preventive measures mainly focus on scalp cooling. This is done either by procedures in which the cooling agent (ice cap or gel cap) must be changed several times or by continuous cooling of the scalp with cold air or cold liquid. There are two scientific rationales for scalp cooling. The first is vasoconstriction, which reduces the blood flow to the hair follicles during peak plasma concentrations of the chemotherapeutic agents and so reduces cellular uptake of these agents. The second rationale is reduced biochemical activity, which makes hair follicles less susceptible to the damage of chemotherapeutic agents. The latter may be more important than vasoconstriction [12]. However, further studies are needed to evaluate and validate the evidence in this field.

One of the objectives of the “Corpo Ritrovato” Scientific Board is to intensify the research on the prevention of alopecia in oncology through topical, oral, and/or mechanical resources.

The use of a wig by the cancer patient remains a valid aid in case of hair and pigmentation trophic changes.

The wig should be free of adhesives, since these can be irritating and sensitizing and with a texture suitable to the needs of a sensitized and altered skin, as occurs during chemotherapy.

Often the use of biological drugs does not lead to alopecia proper, but to massive hair loss which, however, does not cause baldness. In these cases, lotions supplying active agents to hair bulb and stimulating the skin microcirculation may be necessary.

Supplements of antioxidants and substances dedicated to the restoration of the physiological cycle of keratin (vitamin E, melatonin, reduced glutathione, and other active agents in the course of study) can be associated.

Hair dyes based on paraphenylenediamine are not recommended due to hair weakening while hair dyes based on vegetable substances (although they are never 100%) can be used as they cause only a minor contact sensitization in these patients.

Permanent hair waves or pulling hair are not recommended because of the resulting mechanical and chemical stress (powerful oxidation).

### 4.4. Radiodermatitis

Radiodermatitis usually occurs when the skin is exposed to higher doses than 2000cGy, while the dryness associated with flaking and itching is a frequent event. Dermocosmetic care for this event is the same as that described for the control of cleansing and hydration. The dosage specifies the application of cosmetic agents immediately before and immediately after exposure to radiation therapy.

 A new series of antioxidants combined together in order to form real dedicated cosmetics (holly, red vine, ubidecarenone, Q10, extracts of particular seaweed) are very interesting.

### 4.5. Skin Rash and Folliculitis

Skin rash and folliculitis can be considered real diseases rather than beauty flaws. In some cases, the effectiveness of the antineoplastic agent is higher if it produces folliculitis. In some tragic cases, the patient, suffering from and distressed by this skin reaction, suspends the therapy with the risk of not controlling the cancer disease. In this event, the dermatologist plays a decisive role, managing the disease and, at the same time, allowing oncologists to continue the therapy.

The affected skin should be cleansed gently and without astringent agents, and it should be hydrated since xerosis always accompanies folliculitis.

We have already mentioned unsaponifiable substances of karité, jojoba, and olive oil, but there are also sesame, macadamia, and argan oils that, in addition to their hydrating power, also have anti-inflammatory action. 

Topical antibiotics (gentamicin, clindamycin in combination with zinc oxide, mupirocin, and erythromycin) must be chosen in greasy formulations (ointments or creams) and used alternately with a daily dose. If the inflammatory component is evident, a 1% hydrocortisone ointment can be recommended in combination for the first days of therapy.

During the skin reaction, dresses made of special anti-inflammatory action fibers, usually recommended for allergic or atopic patients, can be helpful.

In the presence of overinfection the use of oral antibiotics may be necessary (e.g., tetracycline: doxycycline 200 mg/day).

Chemotherapy must be completely suspended in severe cases with massive exfoliative and bullous components. 

Even in this case, if dermatological therapy is used rapidly with oncologic treatment, the damage can be significantly contained and the patient can complete pharmacological cycles.

### 4.6. Scars

We can apply a cream made of hyaluronic acid, musk rose, and hypericum oil 1-2 times a day on postsurgical scars, so as to improve the elasticity of the tissue and to reduce inflammation. The use of creams, formulated with allium bulb stock extract, makes scars softer and smoother.

Radiotherapy, which is often performed on the scar area, paradoxically improves the scar's cosmetic appearance, causing atrophy. Thus, the scar area must be controlled through careful hydration and photoprotection.

The choice of garments to wear and the detergents to prefer is an important detail to protect a weak skin from irritant and external insults. The garments to wear on skin are completely natural (cotton, linen, silk) and with vegetable colors. Synthetic and elastic garments, rough wool, or clothing with metal and sequins should be avoided.

### 4.7. Photoprotection

both radiation therapy and some chemotherapy medications (5-fluorouracil, vinblastine, dacarbazine) can cause photosensitivity, so it is important to be careful to protect skin during treatment. Direct sunlight during midday must be avoided, and hats and umbrellas should be used to shelter yourself. Tanning beds must be avoided. Some sunscreens can contain irritating chemicals and for this reason sun block should be preferred, as zinc oxide, for maximum protection. We should choose physical sun filters, applied 1-2 hours before sun exposure (the chemical filters may occur contact sensitization). Fatty compounds and formulations should be avoided for their occlusive potential and the risk of developing reactive folliculitis. Treatments that may be recommend are local corticosteroids and emollient lotions for itching. If a reaction is severe, systemic corticosteroids may be prescribed.

## 5. Conclusions

The chance of surviving cancer is both an achievement and a reality of our time. Currently, the synergy between early diagnosis and various combined and personal therapies (surgery, radiation, chemotherapy) has reduced mortality in a significant number of cases, but this is not always accompanied by a satisfactory life quality.

The objective of the project “Corpo Ritrova,to” a scientific board for research and studies on dermocosmetological skin care of oncologic patients, is to give new life to a ruined body, caring for and protecting it at the same time as it undergoes oncologic therapy. 

Hence, the project objective is to improve the quality of life, health, and beauty for those who might suffer or are already suffering from cancer, and it can represent a further important aid for the oncologic patient. 

In this difficult time, it is essential not to lose control and perception of one's own body and to continue to receive treatments of serious and competent specialists, maintaining a good self-esteem and satisfaction.

## Figures and Tables

**Table 1:** Side effects of chemotherapy.

	Alopecia	Flashing	Itching	Xerosis	Neh	Nail disorder	Rash	Phototox	Stomatitis	Mucositis	Vasculitis	Oral ulcers
Bleomicyn	x	x	x	x	x		x		x			
Bortezomib				X								
Busulfan	x		x				x				x	
Capecitabine												
Carboplatin	x						x					x
Cetuximab			x	x								
Cyclophosphamide	x	x			x	x					x	x
Cisplatin		x										
Cytarabine	x				x						x	
Chlorambucil					x		x					
Dacarbazine		x							x			
Dasatinib		x										
Docetaxel	x	x	x			x	x		x	x		
Doxorubicin		x	x		x	x		x	x			
Epirubicin	x											
Erlotinib												x
Etoposide	x	x					x					
Fludarabine												
Flutamide		x										
Gefitinib												
Gemcitabine			x									
Methotrexate							x	x	x		x	
Mitomycin C							x	x				
Nilotinib												
Paclitaxel	x	X				x	x					
Procarbazine							x	x				
Sorafenib												x
Tamoxifen		x									x	
Topotecan	x								x			
Vinblastine	x											
5-FU	x	X					x	x	x			
6 mercaptopurine											x	
Irinotecan	x					x						
Imatinib												
Vincristine	x											

Neh = neutrophilic eccrine hidradenitis.

**Table 2:** Antineoplastic drugs and alopecia.

Antineoplastic drugs that induce alopecia frequently	Antineoplastic drugs that induce alopecia sometimes	Antineoplastic drugs that induce alopecia usually
Adriamycin	Amsacrine	Capecitabine
Cyclophosphamide	Bleomycin	Carmustine
Daunorubicin	Busulfan	Carboplatin
Docetaxel	Cytarabine	Cisplatin
Epirubicin	5-Fluorouracil	Fludarabine
Etoposide	Lomustine	6-Mercaptopurine
Ifosfamide	Melphalan	Procarbazine
Irinotecan	Methotrexate	Raltitrexato
Paclitaxel	Mitoxantrone	Streptozotocin
Topotecan	Mitomycin C	
Vindesine	ThioTEPA	
Vinorelbine	Vincristine	
